# Pathology of callosal damage in ALS: An *ex-vivo*, 7 T diffusion tensor MRI study

**DOI:** 10.1016/j.nicl.2017.04.024

**Published:** 2017-04-30

**Authors:** Agustin M. Cardenas, Joelle E. Sarlls, Justin Y. Kwan, Devin Bageac, Zachary S. Gala, Laura E. Danielian, Abhik Ray-Chaudhury, Hao-Wei Wang, Karla L. Miller, Sean Foxley, Saad Jbabdi, Robert C. Welsh, Mary Kay Floeter

**Affiliations:** a National Institute of Neurological Disorders and Stroke, National Institutes of Health, Bethesda, MD, United States; b Department of Neurology, University of Maryland, Baltimore, MD, United States; c National Cancer Institute, National Institutes of Health, Bethesda, MD, United States; d FMRIB Centre, Nuffield Department of Clinical Neurosciences, University of Oxford, Oxford, UK; e Department of Psychiatry, University of Michigan, Ann Arbor, MI, United States

**Keywords:** AD, axial diffusivity, ALS, Amyotrophic lateral sclerosis, DTI, diffusion tensor imaging, DWI, diffusion weighted imaging, DW-SSFP, Diffusion Weighted Steady State Free Precession, FA, fractional anisotropy, GFAP, glial fibrillary acidic protein, MD, mean diffusivity, MRI, magnetic resonance imaging, PMI, *post mortem* interval, PSI, scan interval (death to scan), RD, radial diffusivity, SNR, signal to noise ratio, VOI, volume of interest, 7 T MRI, Amyotrophic lateral sclerosis, Microglia, Motor neuron disease, Pathology, Steady-state free precession

## Abstract

**Objectives:**

The goal of this study was to better understand the changes in tissue microstructure that underlie white matter diffusion changes in ALS patients.

**Methods:**

Diffusion tensor imaging was carried out in postmortem brains of 4 ALS patients and two subjects without neurological disease on a 7 T MRI scanner using steady-state free precession sequences. Fractional anisotropy (FA) was measured in the genu, body, and splenium of the corpus callosum in formalin-fixed hemispheres. FA of the body and genu was expressed as ratio to FA of the splenium, a region unaffected in ALS. After imaging, tissue sections of the same segments of the callosum were stained for markers of different tissue components. Coded image fields were rated for pathological changes by blinded raters.

**Results:**

The FA body/FA splenium ratio was reduced in ALS patients compared to controls. Patchy areas of myelin pallor and cells immunostained for CD68, a microglial-macrophage marker, were only observed in the body of the callosum of ALS patients. Blinded ratings showed increased CD68 + microglial cells in the body of the corpus callosum in ALS patients, especially those with *C9orf72* mutations, and increased reactive astrocytes throughout the callosum.

**Conclusion:**

Reduced FA of the corpus callosum in ALS results from complex changes in tissue microstructure. Callosal segments with reduced FA had large numbers of microglia-macrophages in addition to loss of myelinated axons and astrogliosis. Microglial inflammation contributed to reduced FA in ALS, and may contribute to a pro-inflammatory state, but further work is needed to determine their role.

## Highlights

• Diffusion-weighted SSFP and histology were carried out on 4 ALS and 2 control brains.
• The FA of the splenium, an unaffected region, was a control for differences in PMI.
• The FA body/FA splenium was reduced in ALS compared to controls.
• The body of the callosum had increased CD68 + activated microglia and astrogliosis.

## 1 Introduction

Diffusion tensor imaging is a tool to evaluate diffusion properties in white matter (Basser, 1995, Pierpaoli et al., 1996) in living subjects, both qualitatively and quantitatively (Pierpaoli and Basser, 1996). Many studies have described changes of white matter diffusion parameters in patients with amyotrophic lateral sclerosis (ALS) (Ellis et al., 1999) which are thought to be caused by loss of integrity of axons undergoing degeneration (Song et al., 2003). A decline in the fractional anisotropy (FA) of the corticospinal tract is the most consistent finding in ALS (Agosta et al., 2010, Ciccarelli et al., 2009, Foerster et al., 2013) although decreased FA also occurs in the body of the corpus callosum (Filippini et al., 2010, Iwata et al., 2011). ALS patients with low FA of the corticospinal tract have shorter survival and more rapid progression (Agosta et al., 2010, Menke et al., 2012). Tissue changes thought to account for changes in diffusion measures in ALS patients are based on animal models that caused reduction in FA values by experimental manipulations that cause axonal degeneration or demyelination (Song et al., 2003, Thiessen et al., 2013). However, other tissue changes might also produce changes in diffusion measures. To date, there are few studies correlating changes in diffusion measures with tissue histology in neurodegenerative diseases.

Over the past ten years, techniques to obtain diffusion imaging in postmortem brains have greatly improved: higher magnetic fields, stronger gradients, signal-to-noise (SNR) optimization and better shimming techniques, among other factors, have allowed imaging of *ex-vivo* human brain tissue at high resolution. New MRI steady-state free precession (SSFP) pulse sequences provide superior diffusion weighted imaging (DWI) of postmortem brain tissue (Buxton, 1993, Foxley et al., 2014, McNab et al., 2009, Miller et al., 2012), compared to classical, spin echo DWI methods (Stejskal and Tanner, 1965, D'Arceuil and de Crespigny, 2007, Pfefferbaum et al., 2004). DW-SSFP methods allow a detailed view of the white matter architecture, as well as quantitative analysis of diffusivity parameters. Although tissue fixation decreases the mean diffusivity (MD) of tissue, FA values are thought overall to remain unchanged over a range of fixation times (Guilfoyle et al., 2003, Sun et al., 2005). *Post mortem* interval (PMI; interval from death to fixation) significantly affects diffusivity measures (Foxley et al., 2014, D'Arceuil et al., 2007). In an animal study comparing 1-, 4-, and 14-day PMIs to immediate fixation, all diffusivity measures in white matter declined with increasing delay of fixation: axial diffusivity (AD) declined most rapidly by 1 day PMI, FA was relatively unchanged at 1-day PMI, but exhibited decline between the 1- and 4- day PMIs (D'Arceuil and de Crespigny, 2007). Consequently, the absolute FA values of postmortem human brains are not directly comparable to *in vivo* imaging.

The goal of this study was to better understand the changes in tissue microstructure that underlie white matter diffusion changes in ALS patients. To accomplish this, we carried out DW-SSFP imaging of postmortem brains of ALS patients and subjects with no known history of neurological disease in a 7 T scanner. The corpus callosum was examined histopathologically. The corpus callosum was chosen for analysis because anatomical segments are differentially affected in ALS, and can be easily identified in different subjects. DTI changes occur in ALS in the body of the corpus callosum and occasionally in the genu, but the splenium is unaffected (Filippini et al., 2010, Iwata et al., 2011). To control for potential differences in PMI across different brains, the FA of the genu and the body of the corpus callosum were expressed as ratios to the FA of the splenium within each subject. Histologic changes that might explain the abnormal diffusion parameters, such as gliosis, inflammation or axonal degeneration, were analyzed qualitatively and semi-quantitatively by blinded ratings of the histological material.

## 2 Methods

### 2.1 Subjects

Six cerebral hemispheres (five males, one female; aged 43–79 years) were obtained from the National Institutes of Health (Bethesda, MD) and from the University of Maryland Brain and Tissue Bank (Baltimore, Maryland) for imaging studies. Informed consent for brain donation was obtained prior to death or from the next of kin. Brains were extracted en-bloc from the skull, hemisected and immersed in 10% formalin (mean postmortem interval, 19.3 ± 10.1 h; range 6–31 h). Brains were stored in formalin at room temperature during the fixation period. Histological studies were carried out on five of the hemispheres, comprised of 4 ALS patients (subjects #3 to #6) and 1 control with no known neurological disease (subject #1). Histology was not carried out for one control hemisphere (#2) because of concerns that the markedly longer fixation time would affect the immunostaining. The mean age of the five subjects with imaging and histology was 63.6 ± 13.9 years. The interval from death to scanning (PSI) ranged from 4 to 10 weeks for these subjects. All ALS patients met revised El Escorial Criteria (Brooks et al., 2000) for definite ALS. Two ALS patients (subjects #5 and #6) carried the C9orf72 hexanucleotide expansion mutation (Renton et al., 2011). A premortem DTI scan had been done on one ALS patient (#6) on a 3 T scanner. Clinical information is summarized on Table 1.

**Table 1:** Summary of demographic data.

Subject	Age	Gender	Diagnosis	C9orf72	Disease duration (months)	PMI (hours)	PSI (days)	Histology
1	43	M	Control	−	−	12	46	+
2	53	M	Control	−	−	24	9 years	−
3	79	F	ALS	−	11	14	49	+
4	57	M	ALS	−	−	31	30	+
5	70	M	ALS	+	24	29	71	+
6	69	M	ALS	+	48	6	34	+

PMI: Postmortem interval (*i.e.* time from death to fixation). PSI – interval from death to scan.

### 2.2 Imaging methods

#### 2.2.1 Specimen preparation for imaging

The surface of the hemispheres was briefly rinsed with a few hundred mls of phosphate buffered saline before the hemisphere was placed in a Plexiglas container filled with Fomblin (Solvay Solexis, NJ), a low proton- fluid which has no MRI signal (D'Arceuil et al., 2007). Air bubbles were removed with vacuum suction, facilitated with periodical gentle shaking of the container for 24 h before scanning.

#### 2.2.2 MRI acquisition

Hemispheres were imaged using a 7 T MRI scanner (Magnetom Siemens, Erlangen, Germany), which has a gradient strength of 70 mT/m and a slew rate of 200 T/m/s with a 32 channel receiver coil. All acquisitions for each hemisphere were obtained in the same scan session. Scanning was performed at room temperature for all specimens. B1 maps based on the Bloch-Siegert approach (Duan et al., 2013) were acquired at the beginning of the sequence protocol to help obtain accurate FA and mean diffusivity (MD) measurements. A 3D balanced SSFP pulse sequence (TE 3.8 ms, TR 7.58 ms, flip angle 35°), which has previously successfully been applied to *ex-vivo* human brain tissue, was used to achieve gray-white matter differentiation (Buxton, 1993, Foxley et al., 2014, McNab et al., 2009, Miller et al., 2011). Four structural 3D balanced SSFP pulse sequences were acquired, divided in two pairs, each with two radiofrequency phase cycling increments of 0° and 180° (Miller et al., 2012). Pairs of balanced SSFP images were acquired before and after the DW-SSFP sequences. The two radiofrequency phases in each pair were averaged to reduce susceptibility artifacts. The second pair was used for evaluating tissue motion and scanner drift. T1 maps were derived from inversion recovery 3DFSE data at eight different inversion times. T2 maps were derived from 3D FSE data at eight different echo times.

Diffusion weighted images were acquired with a DW-SSFP pulse sequence (Buxton, 1993) (resolution 1.0 × 1.0 × 1.0 mm). Diffusion weighting was applied in 49 non-collinear directions, with an applied b effective (Foxley et al., 2014) value of 4000 s/mm^2^, gradient amplitude of 56 mT/m and a gradient duration of 15 ms. Matrix size was 180 × 176 × 176, with a TE/TR of 25/34 ms and a flip angle of 30°. A single-line readout was used, as described by Foxley et al. (Foxley et al., 2014) Four low b_eff_ value acquisitions were also obtained. SNR was calculated from the low b_eff_ value acquisitions. The entire protocol was repeated twice, for a total of 106 volumes. The total acquisition time was approximately 23 h for each hemisphere. The pulse sequence parameters are shown in Table 2.

**Table 2:** MRI pulse sequences.

	Resolution (mm)	TE (ms)	TR (ms)	TI (ms)	Phase encoding steps	Pixel bandwidth (Hz/pixel)	Flip angle	Number of averages
B1 map	4 × 4 × 4	11	149	–	63	260	15	2
Balanced SSFP	0.35 × 0.35 × 0.50	3.8	7.6	–	416	296	35	2 without phase cycling; 2 with 180° phase cycling
T1map (IR)	1 × 1 × 1.4	12	1000	31, 62, 125, 250, 500, 850	150	200	180	1
T2map (TSE)	1 × 1 × 1.4	14, 28, 42, 55, 69, 83, 111	1000	–	150	130	180	1
DW-SSFP	1 × 1 × 1	25	34	–	176	80	30	2

SSFP – steady-state free precession; DW – diffusion weighted.

#### 2.2.3 Image processing: diffusion tensor imaging

The TORTOISE software package (Pierpaoli et al., 2010) was used to preprocess all raw diffusion-weighted images, including rigid body registration and eddy current distortion correction. Diffusion tensors were calculated utilizing a modified version of DTIFit from the FMRIB software library (http://www.fmrib.ox.ac.uk/fsl/) (Smith et al., 2004, Woolrich et al., 2009). Data from the T1 and T2 maps, flip angle, B1 map, gradient duration and amplitude were included in the tensor calculation. Diffusion tensor metrics, including FA and AD, and RD were calculated.

#### 2.2.4 Image processing: volume of interest (VOI) analysis

**Fig. 1:**
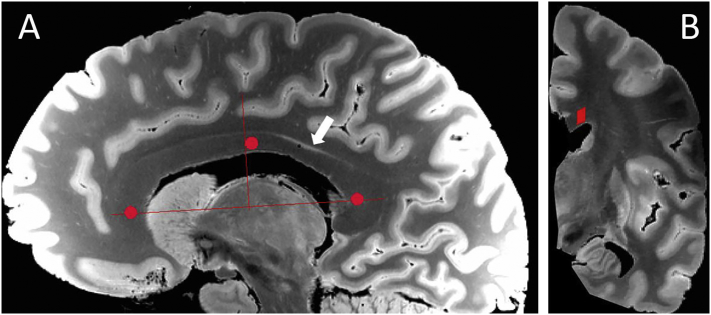
Anatomical acquisitions (balanced SSFP sequence) showing the locations of the volumes of interest (VOI) analyzed (red). A. The anterior-posterior location of the VOIs in the genu, body and splenium of the corpus callosum are shown on a parasagittal image of subject 1. The medial border of the VOI was determined from the most lateral slice in which the lower portion of the cingulate gyrus was still present (white arrow). B. Coronal view of the same hemisphere showing the location of the VOI at the level of the body of the corpus callosum. (For interpretation of the references to color in this figure legend, the reader is referred to the web version of this article.)

FA maps were analyzed utilizing the Medical Image Processing, Analysis and Visualization software package (Bazin et al., 2007). For each scanned specimen, three volumes of interest (VOIs) identical in size were drawn in the genu, body and splenium of the corpus callosum in the sagittal plane of a structural image (Fig. 1A), and subsequently registered to the FA map of the same patient to obtain anisotropy measurements. The first VOI was placed in the midportion of the genu of the corpus callosum. The second VOI was drawn just posterior to the midportion of the corpus callosum, corresponding to the “motor” region in the callosal anatomy classification method described by Hofer and Frahm (Hofer and Frahm, 2006), and to the “posterior mid-body” region according to Witelson (Witelson, 1989). The third VOI was drawn in the anterior aspect of the splenium of the corpus callosum. To create the 3D VOIs, 2D circular ROIs measuring 5 pixels (3.75 mm) in diameter were drawn. These 2D ROIs were then propagated laterally to two additional contiguous images, avoiding the ependymal surface, creating a 5 pixel wide, 3 pixel long cylindrical VOI. Each VOI included an average of 60 voxels. Because analysis was done in hemispheres instead of entire fixed brains, it was not possible to place the VOIs exactly in the midline. To avoid regions of the callosum with potential physical damage from the hemisection, the first 2D ROI was placed in the most lateral parasagittal image in which the inferior portion of the cingulate gyrus was still present (as shown on Fig. 1B).

#### 2.2.5 Method sub-study to validate use of FA ratios of callosal segments

To check whether ratios of FA of the genu and body to the FA of the splenium could be used to control for differences in PMI, thus allowing a comparison among subjects, these ratios were assessed in DTI datasets acquired *in vivo* in 51 scans from healthy controls, and a previously published cohort of 18 ALS patients (Iwata et al., 2011). In the healthy control cohort, the average ratio of FA body/FA splenium was 0.95 ± 0.03, and the average ratio of FA genu/FA splenium was 0.84 ± 0.05. In the ALS cohort, the average FA ratio of body/splenium was 0.85 ± 0.06 and the average ratio of FA genu/splenium was 0.85 ± 0.04 (Supplemental Figure).

### 2.3 Histology

#### 2.3.1 Tissue processing

**Fig. 2:**
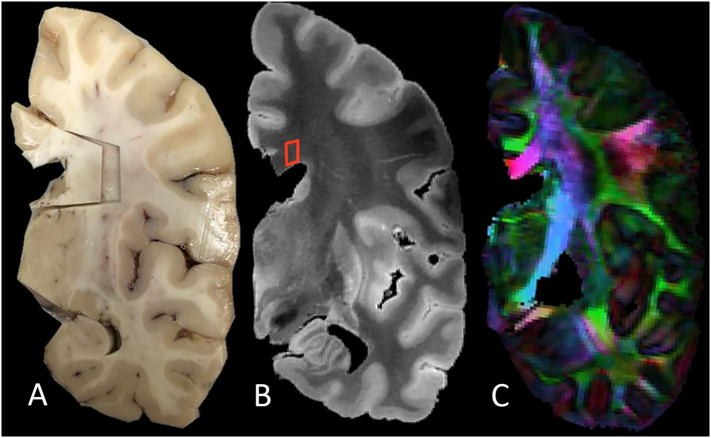
Coronal views of A. the fixed left hemisphere of subject 1 at the level of the midportion of the corpus callosum, showing the *in-situ* block dissected for histological analysis. The tissue block included not only the corpus callosum, but also the cingulate gyrus and part of the caudate nucleus for orientation. B. Anatomical coronal image of the same hemisphere obtained using the balanced SSFP sequence. Red square shows the location of the voxel of interest for FA measures. C. Directionally encoded color map of the same hemisphere, obtained with the DW-SSFP sequence.

**Fig. 3:**
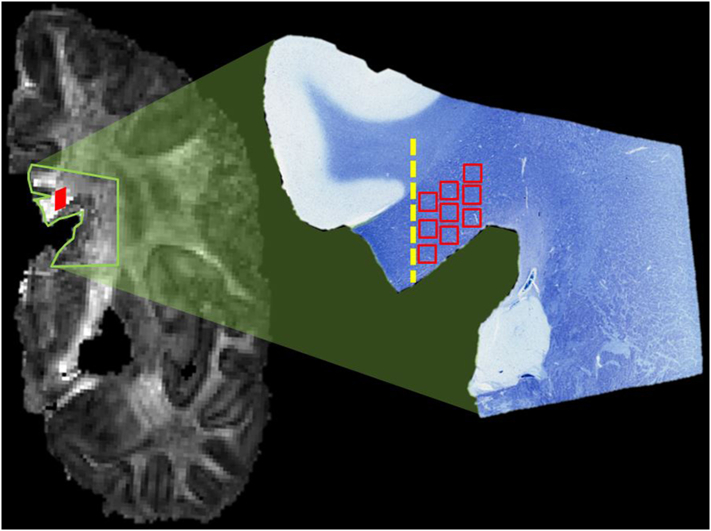
Schematic to illustrate how comparable regions of the brain were analyzed in the diffusion image (coronal image on the left of the figure) and in histological sections (right side, luxol fast blue stained section). Fractional anisotropy was measured in the voxel of interest (filled red square) of the diffusion scan. In the tissue sections, multiple fields of the corpus callosum were photographed in a square grid pattern (open red squares) and coded for analysis. Tissue sections included the cingulate gyrus, which was used as a landmark for the medial border (dashed line) of the grid of fields to be photographed. (For interpretation of the references to color in this figure legend, the reader is referred to the web version of this article.)

After the imaging session, prior to dissection, the midpoint of the callosum was marked with ink on the intact hemisphere. Tissue blocks were dissected from the genu, body and splenium of the corpus callosum in the coronal plane (Fig. 2) and embedded in paraffin. The body was contained in block immediately posterior to the marked midpoint (Hofer and Frahm, 2006). Sections were stained for hematoxylin and eosin, silver stain (Bielschowsky), and luxol fast blue (Fig. 3). Immunohistochemistry was performed using antibodies against CD68, a marker for activated microglial-macrophage cells (Leica Biosystems PA0273 RTU ready to use), GFAP (glial fibrillary acidic protein, Leica Biosystems PA0026 RTU ready to use), an astrocyte marker, and Olig2 (Genetex BTX62440, 1:100), an oligodendrocyte marker. Antibodies were optimized according to each manufacturer's directions. A Leica Bond Max automated stainer was used for histochemical staining.

#### 2.3.2 Qualitative histological analysis

The slides were reviewed by a neuropathologist (A. R-C.) who provided a qualitative assessment of the findings and the adequacy of the staining. The neuropathologist was not blinded to the diagnosis.

#### 2.3.3 Semi-quantitative histological analysis

Digital images were acquired with a Leica Aperio Scanscope (Aperio Technologies, Inc., Vista, California) with 200 × magnification for Olig2, CD68, and GFAP, and with 400 × magnification for Bielschowsky stains. From each section, snapshot images were taken of fields from each corpus callosum segment, in a region extending laterally approximately 3 mm from the lateral edge of the cingulate, with intent to match the general vicinity of the DTI VOI. An average of nine fields were obtained in a square grid pattern from each block (Fig. 3). These images were coded to allow scoring by four different raters who were blinded to the diagnosis and location within the corpus callosum. The raters did not include the neuropathologist who did the qualitative assessment. A total of 500 fields were rated, with 125 fields for each stain.

Each field was scored as follows. CD68 was scored according to presence of positive cells in the field: 0 = none; 1 = mild; 2 = moderate; and 3 = severe (*cf.* Brettschneider (Brettschneider et al., 2012)); GFAP staining in each field was scored for GFAP + reactive astrocytes with enlarged cell bodies and processes: 0 = none; 1 = few; 2 = many; and 3 = field filled with GFAP positive reactive astrocytes and processes. Olig2 was scored as 0 or 1 for presence or absence of evident fascicular organization. Silver stains were scored as 0 or1 according to axonal organization/alignment in bundles and 0 or 1 on the presence of dysmorphic axons (*e.g.* beading or enlargement).

## 3 Results

### 3.1 MRI qualitative analysis

All subjects demonstrated homogeneous signal intensity throughout the CC on the anatomical sequences. FA maps did not depict any obvious areas of signal drop-out. None of the subjects had gross atrophy in the corpus callosum.

### 3.2 DTI quantitative analysis

**Fig. 4:**
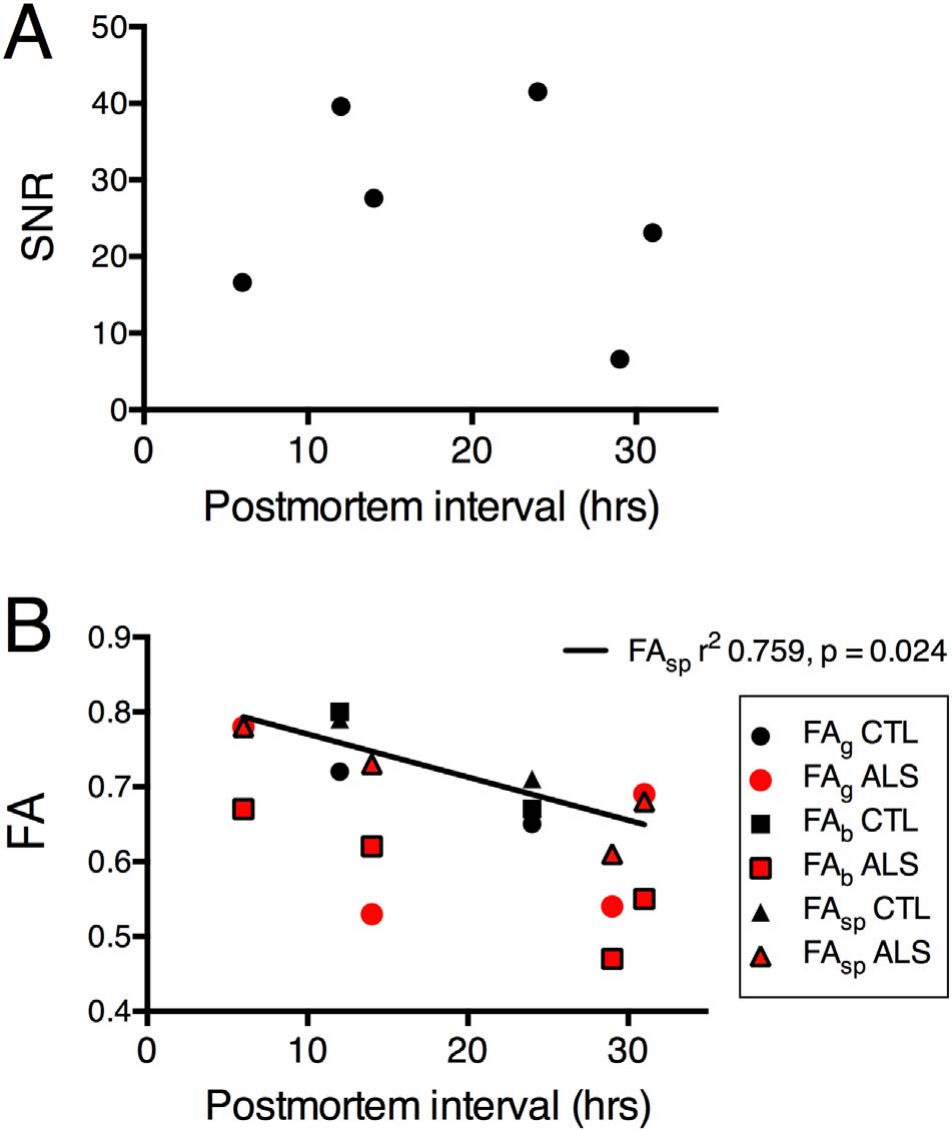
A, B. Effects of postmortem interval on signal/noise ratio (SNR) and fractional anisotropy FA. A. A. There was no evident relationship between the SNR and PMI, which ranged from 6 to 31 h. B. The correlation between PMI and FA was significant only for the VOI of the splenium (FAsp, triangles, solid line). For the two control brains, the values for the FA of the genu (FAg CTL, black circles) and the body of the callosum (FAb CTL black squares) lie close to the values for the splenium. For the four ALS brains, the values for FA of the body of the callosum (FAb ALS, red squares) are lower than the FA values of the splenium (FAsp ALS, red triangles). (For interpretation of the references to color in this figure legend, the reader is referred to the web version of this article.)

The FA measurements in the three segments of the corpus callosum and the FA ratios are shown on Table 3. The FA of the genu and body of the callosum was expressed as a ratio to the FA of the splenium for the purpose of normalization to allow comparison among subjects. The FA body/FA splenium ratios were lower in all ALS patients compared to the 2 control brains. Ratios of 0.86 or lower were observed in all ALS patients and ratios > 0.93 in controls. In ALS, the average of the FA body/FA splenium ratios was 0.82 ± 0.04, whereas in controls, the average ratio was 0.98 ± 0.05. This finding is consistent with the *in-vivo* measurements obtained using a 3 T scanner in 18 ALS patients previously described (Iwata et al., 2011) (FA body/splenium ratio 0.85 ± 0.06) and with measurements from our laboratory's dataset of 51 scans in healthy controls (FA body/splenium ratio 0.95 ± 0.30). The FA genu/FA splenium ratio was similar in ALS patients and controls. Measures of AD and RD were considerably reduced compared to typical measures *in vivo,* with variability between subjects. (Supplemental Tables 1 and 2). The SNR was not correlated with PMI (Fig. 4A), scan interval, or FA. SNR was not significantly correlated with ratios of FA genu/splenium (r^2^ = 0.034, *p* = 0.727) or FA body/splenium (r^2^ = 0.260, *p* = 0.302). PMI exhibited a strong correlation with the FA of the splenium of the corpus callosum of the group of 6 subjects (Fig. 4B; triangles, solid line r^2^ = 0.759, *p* = 0.024). PMI was not correlated with the FA of the genu or body of the callosum, although in the controls (black symbols, Fig. 4B), the values were clustered near the values for the splenium. These findings suggest that the correlation between PMI and FA did not account for the reduction seen in the body or genu of the callosum of the subjects with ALS.

**Table 3:** Mean fractional anisotropy in volumes of interest in callosal segments.

Subject	Diagnosis	Fractional anistropy			Ratio FA genu/splenium	Ratio FA body/splenium	SNR
		Genu	Body (motor)	Splenium			
1	Control	0.72	0.80	0.79	0.91	1.01	39.6
2	Control	0.65	0.67	0.71	0.92	0.94	41.5
3	ALS	0.53	0.62	0.73	0.73	0.85	27.6
4	ALS	0.69	0.55	0.68	1.01	0.81	23.1
5	ALS	0.54	0.47	0.61	0.89	0.77	6.6
6	ALS	0.78	0.67	0.78	1.00	0.86	16.6
ALS	Mean ± SD	0.64 ± 0.12	0.58 ± 0.09	0.70 ± 0.07	0.91 ± 0.13	0.82 ± 0.04	18.5 ± 9.09

FA – fractional anisotropy; SNR – signal to noise ratio.

### 3.3 Histology qualitative analysis

**Fig. 5:**
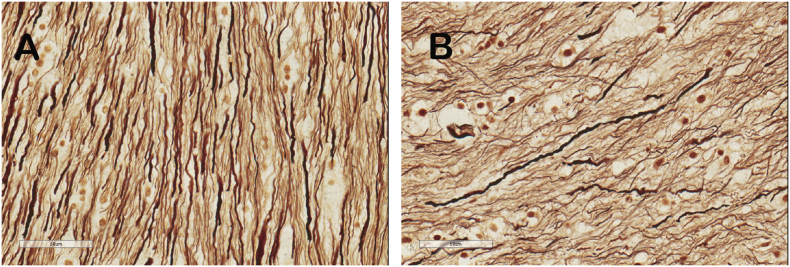
Example of qualitative difference in silver staining of axons in the body of the callosum in the control brain (A) and ALS patient (B, subject 3). The density of fibers was reduced ALS patients in side-by-side comparisons. However, axonal features such as beading and orientation of bundles did not distinguish patients from the control in blinded ratings of image fields.

Sections of the callosal regions of the ALS patients and one control were reviewed qualitatively in an unblinded fashion to assess staining patterns and general pathological findings. In hematoxylin and eosin stained sections, one ALS patient (#4) had mild perivascular lymphocytic infiltration in the genu and body of the callosum, another ALS patient (#3) had mild spongiosis that was noted in the body of the corpus callosum. Luxol fast blue staining demonstrated patchy areas of demyelination in the genu and body of the corpus callosum in several ALS patients. These features were not present in the control case. Qualitative differences in silver staining were seen between the ALS patient and the control subject. ALS patients had patchy areas with reduced silver staining and fragmented axons. Varying degrees of beading and a corkscrew appearance of the axons in the body of the corpus callosum were present both in ALS patients and in the control case (Fig. 5). The quality of the immunostaining for Olig2, CD68, and GFAP, which were used for blinded semi-quantitative analysis by independent raters, was confirmed. ALS cases were noted to have diffuse and patchy areas of increased CD68 immunoreactive microglia-macrophages, accompanied by foamy macrophages. These findings were not seen in the control brain.

### 3.4 Histology semi-quantitative analysis

**Fig. 6:**
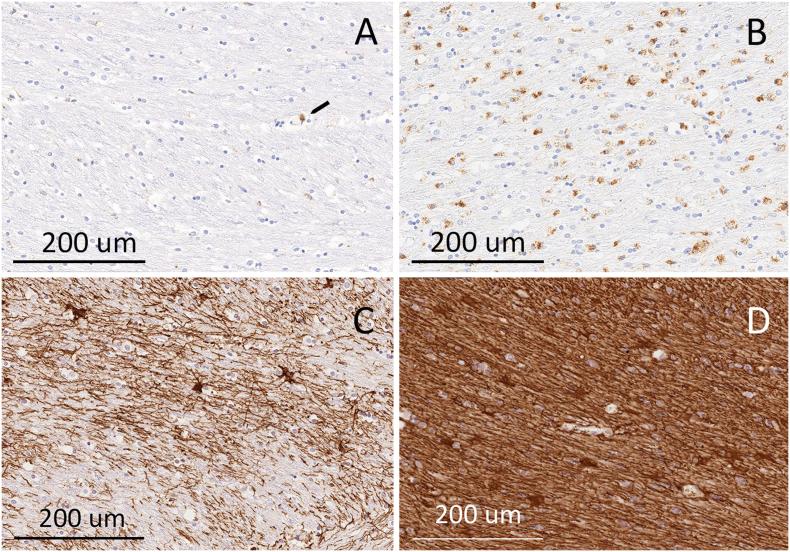
Immunostaining for CD68 (A, B) and GFAP (C, D) in the body of the corpus callosum. A. Control (subject 1) shows only one CD68 + cell, within a blood vessel (black pointer), a normal finding. B. ALS patient (subject 5), shows many CD68 + activated microglia and foamy macrophages in the callosum. C. Control (subject 1) shows sparse GFAP + astrocytes and processes D. ALS patient (subject 6), showing multiple GFAP reactive cells and thickened processes. The histochemical staining of blocks from the control subject and patents 5 and 6 were done at the same time with the same methods.

Table 4 shows the mean score of the blinded ratings from the fields for each callosal segment for each stain. The most consistent difference between ALS patients and the control was seen in CD68 staining. In the control, no CD68 reactivity was seen in the genu or splenium, and two of nine fields in the body of the callosum were graded as having only mild reactivity. Occasional CD68 + macrophages were present in blood vessels in the control and ALS patients (Fig. 6A, black pointer). In the ALS patients the majority of fields in the body of the callosum had increased CD68 + cells; in the two ALS patients with *C9orf72* mutations, CD68 + cells were graded as moderate or severe in all fields of the body of the callosum (Fig. 6A, B). CD68 + reactive microglia and macrophages were more prominent in the body than in the genu or splenium of the callosum. These regions correspond to the VOIs used to assess FA in the diffusion MRI scans.

**Table 4:** Average rating of histological severity of fields from callosal segments by blinded raters.

Callosal segment	Subject	Diagnosis	CD68	GFAP positive cells		Bielschowsky staining		Olig2^a^
				Reactive Astrocytes	Territory overlap	Axon^a^ alignment	Dysmorphic^a^ axons	
Body	2	Control	0.2	0.6	0.3	0.2	0.1	0.6
	3	ALS	0.7	1.8	0.8	0.83	0.7	0.2
	4	ALS	1.0	1.6	0.7	0	0	0.3
	5	ALS, *C9orf72*	2.3	0.9	0.4	0	0	0.3
	6	ALS, *C9orf72*	1.8	1.8	0.7	0.1	0.3	1.0

Genu	2	Control	0	1.2	0.3	0	0	1
	3	ALS	0.3	0	0	0.5	0.4	0.6
	4	ALS	0.4	2.0	1.0	0.3	0.2	0.7
	5	ALS, *C9orf72*	1.8	1.1	0.8	0	0	1.0
	6	ALS, *C9orf72*	2.0	1.7	1.0	0.1	0.1	1.0

Splenium	2	Control	0	0.9	0.4	0	0	0.1
	3	ALS	0.7	0.4	0	0.1	0.6	0.3
	4	ALS	0.4	1.2	0.7	0.3	0.4	0.7
	5	ALS, *C9orf72*	0.5	0.5	0	n.d.	n.d.	0
	6	ALS, *C9orf72*	0.7	1.7	0.8	0.3	0.4	0.3

ALS - amyotrophic lateral sclerosis; CD68 - marker for activated microglia; GFAP - glial fibrillary acidic protein, an astrocyte marker; Olig2 - oligodendrocyte marker. *C9orf72* - carrier of expansion mutation in C9orf72 gene. n.d. - no data obtained.

a Rating scale used was 0 = normal, 1 = abnormal. Other stains rated on a scale of 0, 1, 2.

GFAP + reactive astrogliosis was graded higher in the body of the callosum in ALS patients compared to the control; however, in ALS patients reactive astrogliosis scores were also higher in other segments of the callosum in ALS compared to controls (Fig. 6C, D). Silver staining was scored on two features. Although loss of alignment in parallel bundles was prominent in one ALS patient (subject 3), it occurred in all segments of the callosum and was not seen in the body of the callosum of other ALS patients. Scoring for dysmorphic axons was also marked in this patient, but axonal beading was present to some extent in all subjects. Olig2 staining of oligodendrocyte nuclei was scored according to the presence or absence of the interfascicular pattern typical of organized axonal bundles. There was no consistent disruption in the fascicular organization of Olig2 reactive cells in ALS patients. The Olig2 immunostaining and silver staining suggest that surviving axons in the callosum maintain a fairly normal pattern of alignment.

### 3.5 Comparison of pre-mortem and post-mortem scan

**Fig. 7:**
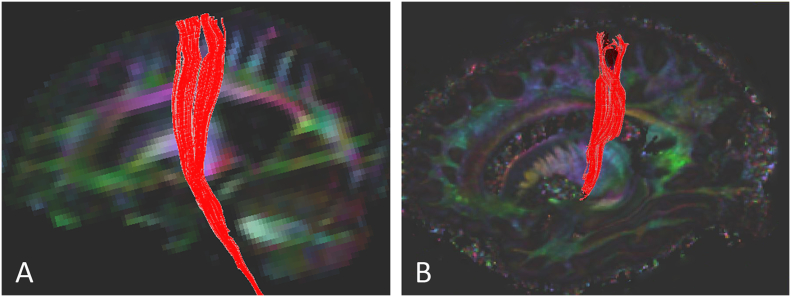
Fiber tracking of the corticospinal tract in subject 6 (ALS) in A. premortem diffusion scan on a 3 T scanner 6 months earlier and B. 7 T DW-SSFP scan of postmortem hemisphere. The corticospinal tract is shown in red in the foreground structure against the directionally encoded color map in the background. Much greater detail can be seen in the 7 T DW-SSFP image, but the corticospinal tract has a similar profile. (Differences in background color-coding result from positioning of the hemisphere in the scanner. The brainstem was not included in this postmortem hemisphere.) (For interpretation of the references to color in this figure legend, the reader is referred to the web version of this article.)

Diffusion tensor imaging had been carried out in one ALS patient approximately 6 and 12 months before death (subject 6), using a 3 T MRI scanner (GE Medical Systems, Milwaukee, WI). In the corpus callosum, the ratio of FA body/FA splenium at both 6- and 12-month premortem scans was 0.86, the same ratio as in the postmortem scan. A side-by-side comparison of the white matter directionally encoded maps of the 6-month pre- and post-mortem DTI scans is shown in Fig. 7, with tractography of the corticospinal tract overlaid in red. Greater anatomical detail of white matter can be appreciated in the high-resolution post-mortem scan. Fiber tracking of the corticospinal tract (DTI Studio software, http://cmrm.med.jhmi.edu) showed a similar configuration in both scans.

## 4 Discussion

In this study, alterations in diffusion imaging measures of white matter in ALS patients that are known to occur with *in vivo* imaging were demonstrated in postmortem brains of ALS patients using a diffusion-weighted steady-state free precession (DW-SSFP) sequence at 7 T (Foxley et al., 2014). We found that the mid-body of the corpus callosum had reduced fractional anisotropy (FA) in diffusion tensor images. The difference in FA between ALS patients and controls was of the same magnitude as seen *in vivo* (see Supplemental Figure). Semi-quantitative histological ratings from the body of the callosum showed increased activated microglia, macrophages and reactive astrocytes compared to a control brain. Qualitatively, the callosum of ALS patients also had loss of myelinated fibers. Based on the patterns of histological changes observed across the different callosal regions and between controls and ALS (Brettschneider et al., 2012), we conclude that the microstructural changes associated with reduced FA in ALS are not solely due to loss of axons or myelin. Infiltration of microglia and astrogliosis contribute to reduced fractional anisotropy. The pathological findings in the corpus callosum are consistent with previous studies (Brettschneider et al., 2012, Sugiyama et al., 2013). Although reduced fractional anisotropy of the corticospinal tract is a hallmark of ALS (Ellis et al., 1999, Agosta et al., 2010, Menke et al., 2012), reduced fractional anisotropy has consistently been found in the body of the corpus callosum (Filippini et al., 2010) where axons from premotor, motor and sensory cortex regions cross (Wahl et al., 2007). Pathological studies have found activated microglia in the corticospinal tract and in the corpus callosum of ALS patients, with more extensive microglial infiltration in ALS cases with *C9orf72* mutations (Brettschneider et al., 2012).

Consistent with previous studies, we found greater numbers of activated microglial in the corpus callosum of ALS patients with *C9orf72* mutations. Animal studies suggest that the *C9orf72* gene product plays a critical role in microglial and macrophage function, such that mice with complete knockout of the gene develop increased inflammation (O'Rourke et al., 2016). Although the expansion mutation in *C9orf72* patients is in a non-coding region, it is possible that haploinsufficiency contributes to increased inflammation in ALS patients with *C9orf72* mutations. Previous PET studies have shown a correlation between ALS disease severity and binding of a radioligand that recognizes activated microglia in the motor cortex and corticospinal tract (Zurcher et al., 2015, Turner et al., 2004). Classically activated microglia have been proposed to play a role in the pathophysiology of ALS by initiating a cascade leading to the release of pro-inflammatory cytokines interacting with inflammatory T-cells (Kuhle et al., 2009, Henkel et al., 2009, Hooten et al., 2015). Astroglia have been shown to play a role in disease progression in transgenic ALS mice (Yamanaka et al., 2008), thought to be secondary to impairment of astrocyte supportive functions or by exacerbating inflammation (Sun et al., 2015). Although astrogliosis was present in the corpus callosum of ALS patients in this study, unlike microglial infiltration, it occurred more diffusely, and did not have greater predominance in callosal segments with reduced fractional anisotropy.

Although degeneration of the corticospinal tract is a hallmark of motor neuron disease, the corpus callosum was chosen for this study because the body is consistently affected in ALS (Filippini et al., 2010), it is straightforward to compare similar segments in different subjects, and has good fixation in hemisected brains. The corticospinal tract was deemed less favorable because the degree of degeneration varies along its proximal-distal axis and it is more difficult to identify the same level in coronal sections obtained at brain cutting. Additionally, penetration of fixative into the depths of the hemisphere where the corticospinal tract lies occurs more slowly, effectively lengthening the postmortem interval. (Miller et al., 2011, Yong-Hing et al., 2005) Diffusivity measures are affected by postmortem interval and the duration of fixation. Mean and axial diffusivity decline rapidly after death, particularly in white matter tracts (D'Arceuil and de Crespigny, 2007). Mean diffusivity also declines with the duration of formalin fixation (Sun et al., 2005). Although FA also declines with increasing postmortem intervals (Miller et al., 2011), the slope is more gradual, and FA exhibits relatively little change with increasing fixation time (Foxley et al., 2014, Guilfoyle et al., 2003, Sun et al., 2005, D'Arceuil et al., 2007). The relative stability of FA measures in fixed postmortem tissue was one rationale for focusing on FA in this study. Despite the relatively narrow range of postmortem intervals in this study, an effect of postmortem interval on FA was observed. The correlation between postmortem interval and FA was evident in the six cases studied only for the splenium, a region of the corpus callosum that is spared in ALS (Filippini et al., 2010, Iwata et al., 2011). In the control brains, but not the ALS brains, the FA genu and body of the corpus callosum was similar to the splenium. In the ALS brains, the FA of the body and genu of the callosum was not correlated with postmortem interval, reflecting the contribution of disease-related changes.

There are several limitations of this study. The sample size was limited, in part related to scheduling long scanner times, up to 23 h in this study, timed to the availability of brains with similar postmortem and fixation intervals. We were only able to carry out a histological evaluation on one of the control brains, but the finding of few activated microglia and little astrocytosis was consistent with an earlier pathological study that included controls without neurological disease (Brettschneider et al., 2012). It was surprising that the semi-quantitative scoring of axonal morphology and organization failed to show differences between the ALS and controls because differences were noted in the unblinded qualitative analysis. The features selected for blinded scoring in the silver stained sections — axonal alignment and morphology — occurred to a similar extent in the control and ALS brain. A possible explanation is that hemisection of the brain at autopsy caused abrupt changes in axons, such as beading, that are seen in acute injury (Skinner et al., 2015), or that beading occurs as an artefact of immersion fixation. Alternatively, other histological methods, for example in semi-thin plastic sections, may have allowed a more sensitive quantification of loss of axon numbers or diameter. Because some brains were obtained from the brain bank, clinical information was limited, particularly regarding the site of disease onset and presence of cognitive involvement in the ALS patients. When scoring of the severity of motor impairment was available, it was typically carried out several months prior to brain donation. Thus, although this study demonstrates pathological changes in the callosum correlated with diffusion measures, in both sporadic and familial ALS, it is possible that study of a larger sample would reveal additional heterogeneity.

The white matter anatomy of the corpus callosum is known to be heterogeneous, with differing proportions of large diameter (> 5 μm) and small diameter(< 0.4 μm) axons in different segments (Aboitiz et al., 1992). Ozturk and colleagues found the highest values of FA in the genu and splenium in healthy controls with slightly lower values in the body of the callosum (Ozturk et al., 2010). Differences in fiber composition may explain the variation in the FA values along the corpus callosum. Using the FA of the splenium for inter-regional normalization the same pattern was found between ALS patients and controls *in-vivo* as *ex vivo.* Although we believe that calculating FA ratios is a reasonable approach in order to normalize for differences in postmortem interval, at the same time we acknowledge that this is an assumption. For this reason, subject 6 was of particular interest in our study. Despite differences in the MRI parameters and the measuring techniques between the premortem and postmortem scans, it is remarkable that the ratios of FA body/splenium were in agreement.

The DW-SSFP sequence was first described by Buxton in 1993 (Buxton, 1993) and was successfully applied to intact fixed human brains by McNab and colleagues (McNab et al., 2009, McNab and Miller, 2008). This technique is particularly useful for evaluation of fixed human brain tissue. First, it has excellent SNR efficiency (Miller et al., 2012). The classic spin echo DWI (Stejskal and Tanner, 1965) used *in vivo* has a tradeoff between echo time and b value, which, ultimately, implies a tradeoff between SNR and diffusion contrast. In contrast, DW-SSFP allows acquisitions with much shorter echo times, making it possible to apply high b values without causing as much T2 signal decay. DW-SSFP does not have a well-defined b-value due to the contribution of a plurality of spin and stimulated echoes with a range of diffusion times. Nevertheless, it is still possible to quantify anisotropy in DW-SSFP provided the signal is modeled properly, including the B1, T1 and T2 values for each voxel (Foxley et al., 2014, McNab et al., 2009). These maps were obtained and the calculation of diffusion measures were accomplished here using the modified DTIFit toolbox (http://www.fmrib.ox.ac.uk/fsl/) (Smith et al., 2004, Woolrich et al., 2009). Secondly, because the DW-SSFP is highly sensitive to motion, it is highly suitable for postmortem specimens. In our study we collected the DTI data applying a single-line readout, which has previously been shown to improve SNR efficiency (Foxley et al., 2014), but has the disadvantage of increasing scanning times. It is challenging to acquire DTI data with high SNR and a small voxel size in large-bore clinical scanners. DW-SSFP has the ability to provide both, using standard clinical scanners.

### 4.1 Concluding remarks

Although reduced fractional anisotropy in the callosum has been reported in several DTI studies of living ALS patients (Filippini et al., 2010, Iwata et al., 2011, Kwan et al., 2012), and pathology of the callosum has been studied (Sugiyama et al., 2013), the current study has combined diffusion imaging with pathology. Postmortem diffusion imaging provides the opportunity to examine the microstructural basis of changes in fractional anisotropy. We have shown that the regional reduction of fractional anisotropy in the callosum of ALS patients can be demonstrated in postmortem brains of ALS patients using a diffusion-weighted steady-state free precession (DW-SSFP) sequence (Foxley et al., 2014). By expressing fractional anisotropy of the body of the callosum as a ratio to the fractional anisotropy of the splenium, the difference between ALS patient brains and control brains was of comparable magnitude to the difference in *in vivo* DTI studies. The histological changes in the region of reduced fractional anisotropy consisted of axonal loss, astrocytosis and microglial infiltration. Microglial infiltration was most prominent in two ALS patients who were carriers of a mutation in the *C9orf72* gene. This study demonstrates the feasibility of combining postmortem diffusion with pathology of selected brain regions, and raises attention to a potential role of microglial activation in degeneration in ALS. Future studies will be needed to assess whether changes in white matter fractional anisotropy *in vivo* precede or follow microglial activation, such as by combining microglial PET (Zurcher et al., 2015) and diffusion tensor MRI.

The following are the supplementary data related to this article.

**Supplemental Figure:**
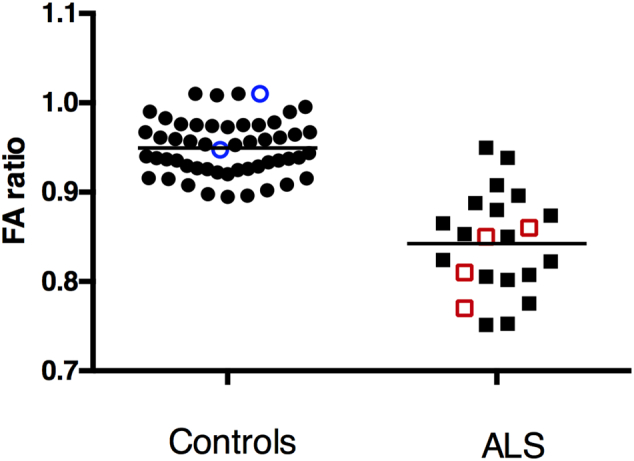
The ratio of the fractional anisotropy of the body/splenium was calculated from 53 *in vivo* 3 T DTI scans of healthy controls (black circles) and 18 sporadic ALS patients (black circles) previously published (Iwata et al., 2011). The FA ratios for the 2 *ex vivo* controls (blue open circles) and the 4 *ex vivo* ALS patient brains (red open squares) obtained in this study are shown on the same plots as the *in vivo* measurements.

Additional diffusion indices for volumes of interest. Image 1

## Disclosures

The authors have no conflicts of interest to disclose.
