# Supplementary material for: Pathology of callosal damage in ALS: An ex-vivo, 7 T diffusion tensor MRI study
Source: Neuroimage Clin. 2017 Apr 30;15:200–8. doi: 10.1016/j.nicl.2017.04.024 (PMC5429246; doi:10.1016/j.nicl.2017.04.024)
Supplement: Supplementary file 1 — Additional diffusion indices for volumes of interest. [file mmc1.docx]

Table S1. **Mean Radial Diffusivity (RD) in Volumes of Interest in Callosal Segments**

|  |  | RD x 10^-3^ mm^2^/s | | |  |  |
| --- | --- | --- | --- | --- | --- | --- |
| Subject | Diagnosis | Genu | Body | Splenium | Ratio genu/  splenium | Ratio body/  splenium |
| 1 | Control | .0167 | .0164 | .0106 | 1.57 | 1.55 |
| 2 | Control | .0438 | .0379 | .0374 | 1.17 | 1.01 |
| 3 | ALS | .0743 | .0554 | .0463 | 1.61 | 1.20 |
| 4 | ALS | .0238 | .0287 | .0209 | 1.14 | 1.37 |
| 5 | ALS | .0306 | .0286 | .0261 | 1.17 | 1.09 |
| 6 | ALS | .0178 | .0291 | .0208 | 0.86 | 1.40 |
|  |  |  |  |  |  |  |
| Mean ± SD | Control | .0302 ± 0192 | .0271± 0152 | .0240 ± 0190 | 1.37 ± 0.29 | 1.28 ± 0.38 |
| Mean ± SD | ALS | .0366 ± 0257 | .0354 ± 0133 | .0285 ± 0121 | 1.19 ± 0.68 | 1.27 ± 0.15 |

Table S2. **Mean Axial Diffusivity (AD) in Volumes of Interest in Callosal Segments**

|  |  | AD x10^-5^ mm^2^/s | | |  |  |
| --- | --- | --- | --- | --- | --- | --- |
| Subject | Diagnosis | Genu | Body | Splenium | Ratio genu/  splenium | Ratio body/  splenium |
| 1 | Control | 0.0821 | 0.0846 | 0.0614 | 1.34 | 1.38 |
| 2 | Control | 0.1712 | 0.1667 | 0.1486 | 1.15 | 1.12 |
| 3 | ALS | 0.2108 | 0.1651 | 0.1764 | 1.20 | 0.94 |
| 4 | ALS | 0.0875 | 0.0811 | 0.0745 | 1.17 | 1.09 |
| 5 | ALS | 0.0795 | 0.0739 | 0.0598 | 1.33 | 1.24 |
| 6 | ALS | 0.0907 | 0.0756 | 0.0786 | 1.15 | 0.96 |
|  |  |  |  |  |  |  |
| Mean ± SD | Control | .1267± .0630 | .1256± .0580 | .1050 ± .0617 | 1.25 ±0.13 | 1.25 ±0.18 |
| Mean ± SD | ALS | .1171 ± .0626 | .0989 ± .0442 | .0973 ± .0533 | 1.21 ±0.08 | 1.05 ±0.14 |
